# Utilising benefit-risk assessments within clinical trials—a protocol for the BRAINS project

**DOI:** 10.1186/s13063-021-05022-0

**Published:** 2021-01-19

**Authors:** Nikki Totton, Steven Julious, Dyfrig Hughes, Jonathan Cook, Katie Biggs, Lizzie Coates, Andrew Cook, Catherine Hewitt, Simon Day

**Affiliations:** 1 School of Health and Related Research, University of Sheffield, Sheffield, UK; 2 Centre for Health Economics and Medicines Evaluation, Bangor University, Bangor, UK; 3 Centre for Statistics in Medicine, Nuffield Department of Orthopaedics, Rheumatology and Musculoskeletal Sciences, University of Oxford, Oxford, UK; 4 Wessex Institute, University of Southampton, Southampton, UK; 5 York Trials Unit, Department of Health Sciences, University of York, York, UK; 6 Clinical Trials Consulting & Training Limited, Buckingham, UK

**Keywords:** Benefit-risk, Randomised controlled trials, Health technology assessment

## Abstract

**Background:**

Depending on the treatment to be investigated, a clinical trial could be designed to assess objectives of superiority, equivalence or non-inferiority. The design of the study is affected by many different elements including the control treatment, the primary outcome and associated relationships.

In some studies, there could be more than one outcome of interest. In these situations, benefit-risk methodologies could be used to assess the outcomes simultaneously and consider the trade-off between the benefits against the risks of a treatment.

Benefit-risk is used within the regulatory industry but seldom included within publicly funded clinical trials within the UK. This project aims to gain an expert consensus on how to select the appropriate trial design (e.g. superiority) and when to consider including benefit-risk methods.

**Methods:**

The project will consist of four work packages:

1. A web-based survey to elicit current experiences and opinions,
2. A rapid literature review to assess any current recommendations,
3. A two-day consensus workshop to gain agreement on the recommendations, and
4. Production of a guidance document.

**Discussion:**

The aim of the project is to provide a guideline for clinical researchers, grant funding bodies and reviewers for grant bodies for how to select the most appropriate trial design and when it is appropriate to consider using benefit-risk methods. The focus of the guideline will be on publicly funded trials however, the vision is that the work will be applicable across research settings and we will connect with other organisations and committees as appropriate.

## Background

Randomised controlled trials (RCTs) are a rigorous methodology used to compare health technologies on a multitude of outcomes in a real-world setting. Often, the aim of an RCT is to provide evidence that a new health technology (HT) is superior to current practice (superiority trial design). As more HTs come into practice, it is common for current practice to be an existing HT. In these circumstances, it is important that the RCTs show the new HT is equivalent, or at least not inferior, to the existing one on the primary health outcome and this remains the primary outcome of the study (equivalence or non-inferiority trial design). Equivalence suggests the two HTs are “not too different” [1] to each other whilst non-inferiority implies that the “new intervention is ‘not unacceptably worse’ than the intervention used as the control” [2].

Key elements of the study, such as the sample size, are dependent on the selected primary outcome and related trial design. However, particularly with equivalence and non-inferiority designs, there are other important outcomes within the study to consider. Considering multiple outcomes reflects the complexity of policy decisions, where information on clinical effectiveness, safety, cost, convenience and/or time could be considered when choosing whether to implement across the National Health Service (NHS).

Benefit-risk (B-R) methodologies can be used in these situations to consider the trade-off between outcomes and evaluate “whether its benefits outweigh its risks” [3]. This is therefore providing a comparison of competing treatments over multiple outcomes. B-R methodology is already commonly used within the regulatory setting where it is important for regulators to be able to evaluate the benefits of a drug against its harms [4]. In 2007, the European Medicine Agency (EMA) published a report which showed the potential value of existing B-R models and methods [5, 6] and further created decision-making models for use in this area [7].

In the UK, the National Institute for Health Research (NIHR) and the Medical Research Council (MRC) publicly fund RCTs to provide the evidence to inform national policy decisions on HTs. Utilising the B-R methods used in regulatory, drug trials in a publicly funded setting requires additional considerations such as economic outcomes and funder perspective.

There are signs that recent work in this area has begun to influence practice [8]. However, these methods are often considered during the analysis phase of the study and not at the design stage which would help to ensure all appropriate information is collected to complete this work during analysis.

Further work is required to consider when different trial designs are appropriate, evaluate the current practice of using B-R in practice for these situations and gain expert consensus on the appropriateness of B-R in this setting to enable the production of more comprehensive guidance for this context.

## Aims and objectives

The overall aim of this study is to provide consensus-driven guidance for the inclusion of B-R approaches within the design of NIHR/MRC funded RCTs.

This aim will be achieved by completing the following specific objectives:

1. Review current practice of B-R methodology in relation to different trial designs (superiority, equivalence, non-inferiority),
2. Review recommended B-R methodologies and the rationale for use within RCTs,
3. Achieve expert consensus on how to select an appropriate trial design and when to consider implementing the recommended B-R methodologies,
4. Produce guidance to inform the inclusion of B-R methodologies within RCTs for NIHR/MRC trials.

## Methods

The project has been split into four work packages (WPs) to represent the four objectives.

- WP1. A survey of relevant researchers about their experiences of using B-R methodologies within RCTs,
- WP2. A rapid literature review to assess any current recommendations for B-R methodologies,
- WP3. A two-day consensus workshop to gain agreement on the recommended design and methods that could be used in the NIHR/MRC setting,
- WP4. Create a guidance document to aid researchers on designing studies and potentially including B-R methodologies within NIHR/MRC grant applications.

### WP1—survey

A web-based survey of current practice will be conducted using the Qualtrics [9] platform. The main objective of the survey is to elicit current use and initial opinions on the use of B-R methodologies in RCTs (industry or publicly funded). In addition, it will be used to identify any methodological updates currently in progress and understand the information required to be within the final guidance document.

The survey will be sent via email to a range of key stakeholders within clinical trials including representatives of trial funding bodies, experts and researchers known to be working in the field to get a range of experiences.

Summary statistics will be completed on the results from the survey and presented as means and standard deviations or medians and inter-quartile ranges for continuous data and counts and percentages for categorical data. No formal statistical testing will take place on the survey data.

### WP2—literature search

A rapid methodological [10] review will be conducted of the published and unpublished guidance on B-R methodology using a pearl growing technique. Pearl growing is a method of iteratively developing search strategies based on the thesaurus and free-text terms associated with articles of known relevance [11]. This will complement the review completed by the PROTECT group which focussed on B-R in drug development [12] by concentrating on B-R methodologies recommended for use in RCTs.

Eligible material will be published and unpublished guidance and methodological articles in English proposing B-R methodology between 1999 and 2019. This chosen time period was to ensure sufficient article identification without repetition and was supported by the timelines presented by Garrison [13]. Formal search strategies will search MEDLINE and Web of Science starting with key terms and papers known to the research team to identify MeSH headings for the formal search strategy. Informal search strategies will focus on Google Scholar, grey literature, reference and citation tracking [14]. The review will be registered on PROSPERO.

The review will link sources to broad methodological categories with other variables including context, rationale, procedures and author-identified methodological strengths and limitations. No formal, pre-specified assessment of methodological quality will take place with deductive reasoning - that bias could be a formal logical consequence of the proposed method—explicated in the narrative synthesis. We will apply the constant comparison method to extracted data [10]. Narrative synthesis will involve one or more tables of B-R rationale and practices.

The results from the literature search will be coupled with the survey responses (WP1) to produce an overview of current possible methods of B-R, their potential uses in RCTs and any strengths and weaknesses found within the literature related to each one. These findings will then feed into the workshop to facilitate discussion about appropriate B-R methodologies.

### WP3—workshop

To reach consensus on how to select a trial design and when to use B-R methodologies, a 2-day expert consensus workshop will be completed which will include presentations, discussions and use the Nominal Group Technique (NGT) to reach consensus as appropriate [15]. NGT is an interactive multi-stage approach which is designed to combine opinion into group consensus during a structured face-to-face meeting. This facilitates the generation of a wide range of ideas, encourages equal participation, helps to avoid conflict and the possibility of some viewpoints dominating and crucially enables the achievement of a credible solution within a short timeframe.

Elements of the workshop will be:

1. A briefing document which summarised the findings of the survey and literature review (containing all B-R approaches identified) will be sent to workshop participants in advance;
2. Presentations will be used to set the scene of the workshop and give all the necessary background information to the topic. This will from different perspectives, e.g. methodology and application so that all outlooks can be considered in the discussions.
3. Brainstorming round/s—panel members will be asked to record their individual thoughts on elements of the guidance such as designing superiority/non-inferiority studies and using B-R methodologies. A “round robin” sharing of ideas will then be completed to allow identification of all potential approaches, followed by a structured whole group discussion;
4. A preliminary rating round will be completed to gain preferences on design and use of B-R methodologies, the results of which will be considered within a second structured group discussion; and
5. A second, final round of rating will be completed to elicit the final preferences of approaches.

Participants of the workshop will be co-applicants of the project along with additional members identified from the responses to the survey. Participants will be invited to aim for input across the industry, academia, funders and policy makers from within the UK and internationally if felt appropriate.

Key discussions from the workshop will be recorded, transcribed and analysed using thematic analysis [16] supported by the NVivo software. These results will be used to provide further detail to the preferences stated within the NGT.

### WP4—guidance document

Using the information gained in WP1–3 with a focus on results from the workshop, a guidance document will be written to incorporate the opinions and preferences of the expert group. The guidance will describe the methods for selecting a trial design, when it is appropriate to use B-R methods and how this relates to the different trial designs. Any key recommendations about particular methods will also be made as appropriate. Recommendations will be illustrated using RCT case studies.

### Sample size

There will be no limit to the number of participants approached for inclusion in the survey and it is anticipated there will be between 25 and 30 participants at the workshop. Guidance on consensus methodology is not overly prescriptive on sample size. Whilst Murphy et al. (1998) [17] recommend between 6 and 12 participants, it was hoped to accommodate a larger number for this project, in-keeping with previous similar work [18], to ensure that all stakeholder groups are represented.

### Dissemination

The guidance document produced from this project will be reported to the MRC and NIHR as well as other funding bodies and stakeholders. The guidance will be published in an appropriate peer-reviewed journal and presented at relevant conferences.

### Project management

The co-applicants of the study will make up an oversight committee which will provide strategic input to the focus, methodology and outputs of the project. These members have a wide range of experience of clinical trials within academia and the pharmaceutical industry to give a range of perspectives to direct the project. There will be two scheduled meetings with this group before the workshop to provide an update to applicants and get feedback as required.

A working group, consisting of SJ, JC, DH and NT, will manage the day-to-day running of the project with monthly meetings scheduled throughout the duration of the project to keep up to date with progress.

## Discussion

A guideline on selecting trial designs and using B-R methodologies within NIHR/MRC funded clinical trials could help facilitate their application in NHS policy decisions. Our aim is to provide a guideline for clinical researchers, grant funding bodies and reviewers for grant bodies. The focus of the guideline will be on publicly funded trials; however, the vision is the work will be applicable across research settings and we will connect with other organisations and committees as appropriate.

### Trial status

The WP1 survey was open between 21 May 2019 and 31 of July 2019. The WP2 full literature search was completed in July 2019 and extraction completed in August 2019. WP3, the workshop, ran in September 2019 and the results from this and WP4, the guidance document will be completed by April 2020. The current protocol is Version 2 dated 10 May 2019. Recruitment within this project was specific to each stage and the inclusions of other experts’ input to the project have no limit. Although the data from the workshop has been collected, the information to include in the guidance document (the key output from the study) will have input and additional data included, as appropriate, from expert members until saturated and the end date for inclusion within the project will be once the guidance has been submitted and the project closed down (April 2021).

## Data Availability

Not applicable

## Abbreviations

B-R: Benefit-risk
EMA: European Medicine Agency
HT: Health technology
MeSH: Medical Subject Headings
MRC: Medical Research Council
NGT: Nominal Group Technique
NHS: National Health Service
NIHR: National Institute for Health Research
PROTECT: Pharmacoepidemiological Research on Outcomes of Therapeutics by a European Consortium
RCT: Randomised controlled trial
UK: United Kingdom
WP: Work package
